# Neurological Manifestations of COVID-19: A Case Report

**Published:** 2020-11

**Authors:** Majid Malekmohammad, Seyed Mohammad Reza Hashemian, Babak Mansourafshar, Hamidreza Jamaati

**Affiliations:** 1 Tracheal Diseases Research Center (TDRC), National Research Institute of Tuberculosis and Lung Diseases (NRITLD), Shahid Beheshti University of Medical Sciences, Tehran, Iran,; 2 Chronic Respiratory Diseases Research Center, NRITLD, Shahid Beheshti University of Medical Sciences, Tehran, Iran.

**Keywords:** COVID-19 encephalopathy, Central nervous system, Ischemia

## Abstract

A novel coronavirus disease (COVID-19) was reported in Wuhan, China in December 2019 and spread rapidly around the world, causing high rates of mortality and morbidity. This disease is known for its respiratory manifestations. Also, there have been several reports of neurological involvement in patients with COVID-19. In this study, we present a 55-year-old Iranian male patient, who was referred from another medical center with a decreased level of consciousness. Upon admission, only respiratory signs of COVID-19 were observed, but later, some neurological manifestations were also observed, such as an alteration in mental status, disorientation, stupor, and finally coma. In radiological studies, a hemorrhagic encephalopathy pattern was detected. Despite improved oxygenation and alleviation of respiratory symptoms with antiviral and anti-inflammatory therapies, cerebral injuries progressed, and the patient died due to severe brain damage.

## INTRODUCTION

Coronavirus disease 2019 (COVID-19) mostly manifests as pneumonia, involving the lower respiratory tract. This disease may also invade the nervous system. The most common symptoms of COVID-19 include fever, cough, and fatigue, while neurological manifestations, such as seizure, an altered level of consciousness, cerebral ischemia (1–4), and encephalitis may be also seen in some patients. Here, we report the case of a patient with lethal neurological complications.

## CASE SUMMARIES

The patient was a 55-year-old man, referred to our center with complaints of fever, chills, cough, and fatigue over the last four days. Fever, chill, cough, and fatigue were the first manifestations of the disease, but with time, dyspnea also emerged. The patient was admitted to a center with an oxygen saturation of 75%. His medical history indicated diabetes, without any history of cardiovascular disease or hypertension.

During hospitalization in the first center, deterioration of mental status and exacerbation of dyspnea were detected. Accordingly, intubation and mechanical ventilation were applied, and the patient was referred to our center. At the time of admission, the intubated patient was in a coma with no response to painful stimuli; no lateralization or neck stiffness was observed. His vital signs were as follows: temperature of 38.3°C, blood pressure of 115/65 mmHg, pulse rate of 105 bpm, and respiratory rate of 15 bpm. He was transferred to the intensive care unit (ICU) under intubation and was then connected to a ventilator.

The patient’s laboratory test results upon admission were as follows: pH= 7.25; partial pressure of carbon dioxide (PCO_2_)= 39 mmHg; O_2_ saturation= 78%; bicarbonate (HCO_3_)= 19 mmol/L; fasting blood sugar (FBS)= 121 mg/dL; urea= 95 mg/dL; creatinine= 1.0 mg/dL; Na= 146 mEq/L; K= 4.5 Eq/L; Mg= 2.6 Eq/L; Ca= 8.1; phosphate= 2.9 mg/dL; aspartate aminotransferase (AST)= 116; alanine aminotransferase (ALT)= 299; white blood cell count (WBC)= 9720 c/μL; lymphocytes= 7.8%; Hb= 11 g/dL; platelet count= 140,000/μL; procalcitonin level= 0.17; interleukin-6 (IL-6)= 1.7 pg/mL; HbA1C= 7.7; albumin= 2.0 g/L; and C-reactive protein (CRP)= 60 mg/L. During the patient’s hospital stay, therapy was applied with suspicion of COVID-19, despite three negative PCR results. The antiviral therapy included chloroquine (400 mg) daily, along with lopinavir/ritonavir (400/100 mg). However, because of severe involvement of the lungs and hypoxemia, daily intravenous immunoglobulin (30 mg of IVIG) was added to his regimen. Also, broad-spectrum antibiotic therapy was initiated for the patient to evaluate the cause of mental health deterioration. The lumbar puncture indicated WBC=0, red blood cell (RBC) count=250, protein=65 mg/dL (15–45 mg/dL), and adenosine deaminase (ADA)=12 U/L (up to 16 U/L).

Over the next few days, the patient’s mental status and consciousness progressively deteriorated, leading to a Glasgow Coma Scale (GCS) score of four. Brain and lung CT scans were acquired, and an anti-inflammatory treatment was initiated. His neurological status exacerbated despite the improvement of oxygen level, clinical status, and radiological findings of lung involvement. Additional anti-inflammatory therapy, including dexamethasone and interferon β-1a, was applied. The level of IL-6 was measured to start administering blocking agents (1.7 pg/mL).

The cerebrospinal fluid (CSF) was examined for cytology, culture, smear, bacterial profile, biochemistry, and PCR assay of COVID-19. The CSF analysis indicated normal results, while the PCR result of CSF and smear of bronchoalveolar lavage were negative. In the pulmonary CT scan, bilateral infiltrations were detected, and in the brain CT scan, multiple hypodense lesions were observed. At the center of the largest lesion, a hemorrhagic pattern was detected. Deterioration of the patient’s condition continued despite all therapies. Favipiravir was administered for the patient, but he died due to brain damage.

**Figure 1.**
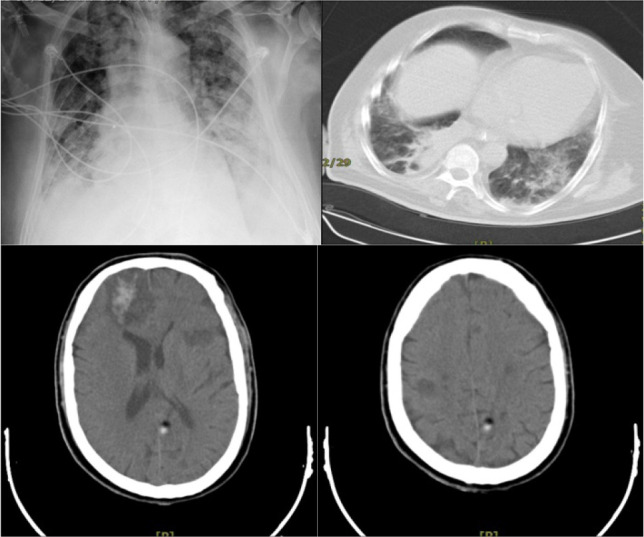
Pattern of pulmonary and brain involvement of patient CT scan

## DISCUSSION

The coronavirus family is categorized as neurotropic microorganisms (5). The viral particles of this family have been found in brain neurons. SARS-CoV-2 appears to be neurotropic, causing neurological symptoms, similar to other members of the coronavirus family (6–8). There are some theories explaining the mechanisms of neurological involvement in COVID-19:

1. Direct invasion of the virus to the nervous system (9, 10);
2. Entry to the nervous system through hematogenous pathways (9, 11);
3. Peripheral nerves, similar to olfactory nerves, can facilitate viral entry (12, 13). They can be a possible route for the virus to enter the central nervous system and may be responsible for anosmia in patients with COVID-19 (14–16);
4. Cerebrovascular events secondary to hypoxia, such as stroke; and
5. The immunological pathway in severe and uncontrolled reactions of the immune system to SARS-CoV-2, systemic inflammatory response syndrome (SIRS), or cytokine storm with inflammatory cytokines, such as IL-6, can cause neurological and pulmonary injuries (17).

Different neurological symptoms have been reported for COVID-19. In a previous study (3), 36% of patients had different neurological manifestations, which can be classified into three major groups: 1) musculoskeletal pain; 2) involvement of the central nervous system; and 3) peripheral nervous system involvement. Symptoms can be as simple as headache, confusion, cognitive impairment, olfactory disorder, impaired taste, vision problems, ataxia, and muscular pain or as severe as seizure, ischemic stroke, demyelination, and encephalopathy (18, 19).

Headache, an altered level of consciousness, paralysis, paresthesia, and seizure (20) are the most common neurological manifestations (12). These signs appear later than respiratory signs (18). In our patient, constitutional symptoms, such as fatigue, malaise, fever, and weakness, along with respiratory signs, such as cough and dyspnea, were the initial manifestations of the disease. In the first week, exacerbation of respiratory symptoms led to the patient’s hospitalization. During his hospital stay, progression of neurological manifestations and deterioration of mental status led to intubation.

In our patient, the most probable cause of brain injury was a hypoxic state for days, although thromboembolic events could be also influential, and we cannot rule out this possibility. Also, immunological events with severe inflammatory responses, cytokine storms, and systemic hemophagocytic lymphohistiocytosis should be considered as possible causes of pulmonary and brain lesions. In a similar study in the United Arab Emirates, a case of COVID-19 with meningoencephalitis and intracerebral hemorrhage was reported (21).

In our study, despite radiological and clinical manifestations of COVID-19 in the patient, the PCR assays of nasopharyngeal sample, bronchoalveolar fluid lavage, and CSF were all negative. According to a study in China on 1014 patients with COVID-19, the sensitivity of RT-PCR assay was estimated at 59%, whereas the sensitivity of pulmonary CT scan was almost 97%, which highlights the importance of clinical and radiological manifestations rather than laboratory tests (22).

In our patient, a CT scan pattern of bilateral ground-glass opacity was noticeable. Nevertheless, several negative PCR results can be confusing. The question arises as to what the possible causes of false negative results can be. According to the published data, up to 20% of RT-PCR results may be false negative (23). The following factors may be influential in the results of PCR assays: 1) How the sample is taken from the patient and how it may affect the genome of the virus; 2) the required amount of the sample; 3) sensitivity of the diagnostic laboratory kit; 3) knowledge and accuracy of companies producing the kits about the unknown virus genomes; and 4) low viral load in the patient (23, 24).

In several studies on patients with SARS-CoV-2 in CSF, there were no indications of CSF infection despite possible neurological manifestations. There are some theories explaining this finding. A latent immunologic response secondary to viremia can be the cause of the nervous system damages. Also, we should consider the low sensitivity of RT-PCR assays, delayed lumbar puncture, collection of fluid, and clearance of virus from CSF (13,25, 25). The manifestations are often gradual and may appear without fever or cough, which can be confusing and lead to misdiagnosis. However, if a radiographic image is acquired from the patient’s lungs, severe lung tissue damage can be seen. In these patients, the PCR results are more likely to be negative (4).

In more advanced stages of the disease, while immunological and inflammatory responses are triggered, systemic angiopathies, thromboembolic events, stroke, and even hemorrhagic encephalopathies may occur (27). In our patient, the only considerable finding of the CSF analysis was the elevated protein content. Also, in similar studies on the CSF of other COVID-19 patients, normal findings were reported (17).

In this case report, our patient receiving appropriate antiviral and anti-inflammatory therapies died secondary to brain injuries, despite the improvement of oxygen saturation and radiological findings of the lungs. In a retrospective study in Wuhan, China on 214 patients, 36% of cases showed neurological manifestations, and this number increased to 49% in more severe cases (3). It should be noted that the nervous system invasion may be a cause of mortality in patients.

## CONCLUSION

It is known that SARS-CoV-2 can invade the nervous system. Although the frequency of respiratory manifestations was higher than neurological manifestations, they may be concomitant and lethal for the patient. Therefore, proper timing of antiviral and anti-inflammatory therapies for patients can improve their overall status and prevent further pulmonary complications. Also, the central nervous system injuries can lead to death and increase the mortality rate of the disease.
